# Evaluating Osteoporosis in Chronic Kidney Disease: Both Bone Quantity and Quality Matter

**DOI:** 10.3390/jcm13041010

**Published:** 2024-02-09

**Authors:** Maria J. Lloret, Maria Fusaro, Hanne S. Jørgensen, Mathias Haarhaus, Laia Gifre, Carlo M. Alfieri, Elisabet Massó, Luis D’Marco, Pieter Evenepoel, Jordi Bover

**Affiliations:** 1 Nephrology Department, Fundació Puigvert, Cartagena 340-350, 08025 Barcelona, Spain; 2 Institut de Recerca Sant Pau (IR-Sant-Pau), 08025 Barcelona, Spain; 3 National Research Council (CNR), Institute of Clinical Physiology, 56124 Pisa, Italy; dante.lucia11@gmail.com; 4 Department of Medicine, University of Padua, 35128 Padua, Italy; 5 Institute of Clinical Medicine, Aarhus University, 8000 Aarhus, Denmark; hsjorgensen@clin.au.dk; 6 Department of Nephrology, Aalborg University Hospital, 9000 Aalborg, Denmark; 7 Division of Renal Medicine, Department of Clinical Science, Intervention and Technology, Karolinska Institutet, Karolinska University Hospital, Huddinge, 141 86 Stockholm, Sweden; mathias.loberg-haarhaus@regionstockholm.se; 8 Diaverum AB, Hyllie Boulevard 53, 215 37 Malmö, Sweden; 9 Rheumatology Department, University Hospital Germans Trias I Pujol, Universitat Autònoma de Barcelona, 08193 Badalona, Spain; lgifre.germanstrias@gencat.cat; 10 Unit of Nephrology Dialysis and Renal Transplantation Fondazione IRCCS Cà Granda Ospedale Maggiore Policlinico, 20122 Milan, Italy; carlo.alfieri@unimi.it; 11 Department of Clinical Sciences and Community Health, University of Milan, 20122 Milan, Italy; 12 Nephrology Department, University Hospital Germans Trias I Pujol, REMAR-IGTP Group, Research Institute Germans Trias I Pujol (IGTP), Universitat Autònoma de Barcelona, 08193 Badalona, Spain; emassoj.germanstrias@gencat.cat (E.M.); jbover.ics@gencat.cat (J.B.); 13 Grupo de Investigación en Enfermedades Cardiorenales y Metabólicas, Departamento de Medicina y Cirugía, Facultad de Ciencias de la Salud, Universidad Cardenal Herrera-CEU, CEU Universities, 46115 Valencia, Spain; luisgerardodg@hotmail.com; 14 Nephrology and Renal Transplantation Research Group, Department of Microbiology, Immunology and Transplantation, KU Leuven, 3000 Leuven, Belgium; pieter.evenepoel@uzleuven.be

**Keywords:** osteoporosis, bone mineral density, DXA, fractures, chronic kidney disease, densitometry, bone quality

## Abstract

Bone strength is determined not only by bone quantity [bone mineral density (BMD)] but also by bone quality, including matrix composition, collagen fiber arrangement, microarchitecture, geometry, mineralization, and bone turnover, among others. These aspects influence elasticity, the load-bearing and repair capacity of bone, and microcrack propagation and are thus key to fractures and their avoidance. In chronic kidney disease (CKD)-associated osteoporosis, factors traditionally associated with a lower bone mass (advanced age or hypogonadism) often coexist with non-traditional factors specific to CKD (uremic toxins or renal osteodystrophy, among others), which will have an impact on bone quality. The gold standard for measuring BMD is dual-energy X-ray absorptiometry, which is widely accepted in the general population and is also capable of predicting fracture risk in CKD. Nevertheless, a significant number of fractures occur in the absence of densitometric World Health Organization (WHO) criteria for osteoporosis, suggesting that methods that also evaluate bone quality need to be considered in order to achieve a comprehensive assessment of fracture risk. The techniques for measuring bone quality are limited by their high cost or invasive nature, which has prevented their implementation in clinical practice. A bone biopsy, high-resolution peripheral quantitative computed tomography, and impact microindentation are some of the methods established to assess bone quality. Herein, we review the current evidence in the literature with the aim of exploring the factors that affect both bone quality and bone quantity in CKD and describing available techniques to assess them.

## 1. Introduction

Patients with chronic kidney disease (CKD) have a higher risk of fractures than the general population, and the incidence of fractures increases as CKD progresses. The incidence of hip fracture across the spectrum of CKD is 2–4 times higher than that observed among people without CKD matched for age and sex. Moreover, hip fractures occur at younger ages, resulting in longer hospitalizations and conferring a higher mortality risk in patients with CKD as compared to their kidney-healthy counterparts [1]. It has been estimated that a patient on hemodialysis (HD) who suffers a hip fracture will do so on average 10 years earlier than the general population [2], confirming CKD as a condition of accelerated aging [3]. Vertebral fractures are very common in both the general population and in patients with CKD, with a similar prevalence. Due to their often asymptomatic nature, vertebral fractures are underdiagnosed. Recognizing vertebral fractures is crucial to identifying high-risk patients and implementing measures to prevent subsequent vertebral and non-vertebral fractures. Worldwide, the prevalence of CKD is >10% [4], and it increases with age (28% among those aged > 70 to 80 years) [5]. Osteoporosis is also frequent in those over 50 years old, so these two conditions are bound to coexist. Moreover, both conditions are set to become increasingly important health problems in our aging populations.

Among patients on HD, mortality after a fracture is 2.7-fold higher than among patients not on dialysis [2]. This increase in mortality is attributable in part to the fact that patients who fracture are more fragile and have more comorbidities and, in part, to the higher rate of complications observed among CKD patients hospitalized for fracture as compared with the general population. CKD patients have higher rates of infections, experience more cardiovascular and cerebrovascular events, are at greater risk of bleeding, and may have less access to surgical treatment due to the complexity of their condition. Moreover, quality of life is substantially reduced after a fracture because of functional loss, chronic pain, and increased polypharmacy. Fragility fractures also represent a high economic cost, with hip fractures being the most devastating [6]. Yet despite the very significant impact that osteoporosis has on patients with CKD, there has been a lack of attention to the most appropriate approach for these patients.

Osteoporosis is currently defined as a decrease in the overall mechanical bone strength, causing an increased risk of low-impact fractures (falls from the patient’s own height) and their deleterious consequences [7]. Bone strength is determined not only by the quantity of bone (mostly determined by bone mineral density [BMD]), measured by dual-energy X-ray absorptiometry (DXA) [8], but also by the bone quality, determined by the microarchitecture and mechanical properties of bone [9]. For instance, the trabecular bone score (TBS) is a practical tool of microarchitecture assessment (available since 2013), among other techniques that will be described thereafter.

Classically, bone fragility in a patient with CKD has been attributed to renal osteodystrophy (ROD), and for that reason, the KDIGO (Kidney Disease Improving Global Outcomes) guidelines in 2009 did not recommend routine BMD testing in CKD G3-5D with the rationale that “BMD does not predict fracture risk as it does in the general population, and BMD does not predict the type of ROD (evidence 2B)” [10]. Subsequently, however, several prospective studies demonstrated that low BMD does correlate with increased fracture risk across the entire spectrum of CKD [11,12]. As new evidence has been introduced, guidelines were updated in 2017 recommending that “in patients with CKD G3a–G5D with evidence of CKD-MBD and/or risk factors for osteoporosis, we suggest BMD testing to assess fracture risk if results will impact treatment decisions (evidence 2B)” [13].

**Figure 1:**
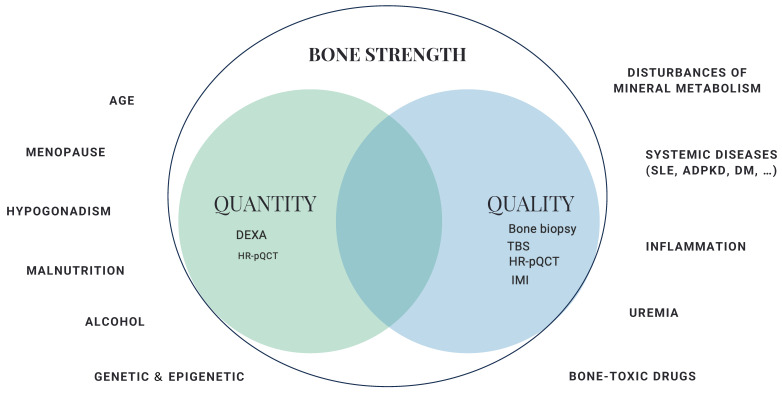
Determinants of bone strength in chronic kidney disease [17]. DXA: dual-energy X-ray absorptiometry; HR-pQCT: high-resolution peripheral quantitative computed tomography; TBS: trabecular bone score; IMI: impact microindentation; SLE: systemic lupus erythematosus; ADPKD: autosomal dominant polycystic kidney disease; DM: diabetes mellitus.

Classical factors associated with osteoporosis that are shared with the general population, including age, lifestyle, nutrition, physical function, genetic, epigenetic, and hormone-dependent factors, should also be considered when assessing bone fragility in a patient with CKD [14]. The coexistence of traditional and non-traditional factors specific to CKD (uremia, acidosis, inflammation, primary kidney disease, etc.) shapes the current concept of “CKD-associated osteoporosis” [15], where it may be especially important to assess not only bone quantity but also bone quality (Figure 1). Although quantification of BMD through DXA is the gold standard for evaluating bone fragility, this may underestimate the risk of fracture in a patient with CKD since a main limitation of this technique is that it essentially measures bone quantity. However, little attention has been devoted to the assessment of bone quality in CKD [16]. The techniques available for evaluating bone quality are not well known, and furthermore, there are some limitations to their application in daily clinical practice. Moreover, although the relevant scientific literature is growing, evidence remains limited, especially in the setting of CKD. For this reason, in this review, we discuss determinants of bone quantity and quality, as well as currently available diagnostic tools and their clinical performance in the setting of CKD. For this review, a comprehensive literature review in PubMed/Medline (updated December 2023) was performed using the terms osteoporosis, BMD, DXA, fractures, CKD, densitometry, and bone quality.

## 2. Bone Quantity

### 2.1. Decreased Bone Mass in CKD

Bone remodeling is a process in which old bone is replaced by new bone, allowing the maintenance of mineral homeostasis and bone strength [18]. It is estimated that renewal of the entire skeleton can take approximately 10 years. This process occurs in remodeling units where the recruitment of osteoclasts leads to bone resorption so that, after apoptosis of these osteoclasts, osteoblasts are recruited, leading to the formation and subsequent mineralization of new bone. The processes of bone resorption and bone formation are coupled in space and time. In young adults, the amounts of bone reabsorbed and formed are similar, i.e., bone remodeling is balanced. Over the years, however, this balance in remodeling is lost, with inadequate formation following resorption, causing a progressive loss of bone, estimated at 0.5–1% per year from middle age onward and accelerating in women after menopause [19].

As 35% of patients with CKD are older than 65 years, loss of bone mass related to aging is expected in this population. However, patients with CKD also present premature aging, and bone loss appears early. In a study that compared 113 patients with CKD (mean glomerular filtration rate [GFR] of 37 mL/min) and 89 age-matched healthy controls, BMD was observed to be markedly reduced in young patients with CKD [20]. Patients with CKD had significantly reduced BMD at the spine (−6.3%), femur (−12.1%), forearm (−5.7%), and whole body (−4.2%) compared with healthy controls. The rate of bone loss is not constant, but it has been observed that patients receiving dialysis experience a loss of 1.2% of BMD at the total hip per year [21].

BMD is influenced both by environmental and genetic factors, the latter being responsible for 50–85% of the normal variability in bone mass [22]. The human skeleton is composed of cortical and trabecular bone, with the former representing up to 80% of the skeletal mass. The main role of cortical bone is to provide mechanical support, whereas trabecular bone also serves an endocrine function. The proportions of the two bone compartments depend on the skeletal site, with a predominance of cortical bone in the hip and mid-radius and trabecular bone in the spine. Secondary hyperparathyroidism causes mainly cortical bone loss [23]. This explains the disproportionally high fracture burden in the peripheral skeleton [24]. Consistently, a small study of 31 patients receiving dialysis who underwent bone biopsy showed that patients with low turnover had more vertebral fractures than patients with osteitis fibrosa, in whom fractures were predominantly appendicular [25]. Thus, it is important to consider not only the total bone mass but also to what extent the different bone compartments (cortical/trabecular) are affected.

Sex hormones are essential for bone health in both women and men. Estrogen deficiency in women is responsible for the rapid loss of BMD and increased incidence of fractures after menopause [26]. Estrogen deficiency leads to increased osteoblast apoptosis as well as increased osteoclastic half-life and activity, leading to a negative bone balance [27]. Hypogonadism also increases remodeling speed, which leads to cortical thinning and porosity [28]. The uremic environment entails alterations in the hypothalamic regulation of gonadotropin secretion, gonadal toxicity, and increased prolactin release. The HELP (Hemodialysis and estrogen levels in postmenopausal) multicenter study revealed that postmenopausal women receiving dialysis have decreased estradiol levels [29]. Early menopause in CKD has been cited as the most underdiagnosed and neglected problem in nephrology [17,30] and is often not even recorded in the clinical history. In a recent Spanish epidemiological study in which data were collected from patients with CKD who had been diagnosed with osteoporosis, the prevalence of early menopause was 9.4% [31], far below the high prevalence described in CKD [32]. Testosterone also plays an important role in bone as it is involved in estradiol aromatization [33]. Testosterone deficiency is observed in 44% of the male dialysis population [34]. It is important to be aware of the potential gender bias in the epidemiology of osteoporosis, as men may be less likely to be screened owing to the perception that they are at a lower risk [35]. This bias may contribute to a potential underestimation of the osteoporosis burden in male patients with CKD.

Chronic metabolic acidosis, a frequent condition in CKD, stimulates osteoclastic activity and inhibits osteoblastic activity, decreasing BMD [36]. Some drugs commonly used in patients with CKD, such as loop diuretics, proton pump inhibitors, unfractionated heparin, vitamin K antagonists, or some antidiabetic drugs [37], can contribute to increased bone fragility via different mechanisms. Glucocorticoids, a mainstay in the treatment of glomerular diseases and renal transplantation, deserve special attention. In corticosteroid-induced osteoporosis, bone resorption is initially increased by enhanced osteoclast differentiation and maturation, and subsequently, osteoblastogenesis is inhibited and osteoblast and osteocyte apoptosis are promoted, resulting in decreased bone formation with long-term use [38].

### 2.2. DXA for Bone Quantity Measurement

DXA is a non-invasive radiographic technique that measures BMD (g/cm^2^) at the total hip, femoral neck, lumbar spine (L1/L2–L4), and distal forearm (ultradistal and distal third of radius). The determination of bone quantity by BMD measurement has long been the gold standard for the assessment of bone strength in the general population, as epidemiological studies clearly demonstrate that the risk of fracture increases as BMD decreases, even in CKD [39]. Prospective cohort studies have shown that BMD is a good predictor of peripheral and hip fractures across the spectrum of CKDG 3-5D [11,12]. Treatment-related BMD changes are strongly associated with fracture reductions, supporting BMD as a surrogate outcome for fracture in future studies [40,41]. In 1994, the World Health Organization concluded that osteoporosis should be defined based on the T-score, which expresses the number of standard deviations of BMD with respect to a young woman aged between 20 and 29 years (maximum peak bone mass) and correlates with an exponential increase in fracture risk. Osteopenia (T-score between −1 and −2.5 SD) and osteoporosis (T-score ≤ −2.5 SD) increase the risk of fracture by two and four times, respectively. Severe osteoporosis is defined as a T-score between −3.5 and −4.5 SD, and established osteoporosis is defined as cases in which a patient has suffered a fragility fracture with a T-score ≤ 2.5 SD. The Z-score represents the deviation of bone mass from the expected value for the patient’s age and sex and is used for the diagnosis of osteoporosis in premenopausal women or men aged < 50 years [42]. Importantly, new criteria for the diagnosis of osteoporosis have been proposed. Briefly, the presence of a hip fracture is currently considered a diagnosis of “osteoporosis,” as well as a non-hip fracture with “only” densitometric osteopenia [43]. Some authors argued that applying the traditional T-score cut-off of −2.5 may not be suitable for all populations. There is a case to be made that populations in specific Asian countries or regions may exhibit distinct risk profiles compared to Caucasian populations. Consequently, diagnostic cut-offs and models for fracture prevalence should be more appropriately tailored to these populations [14].

DXA cannot differentiate between cortical and trabecular bone, as previously mentioned, but the skeletal location can indicate whether there is greater involvement of one type of bone or the other. However, clinical practice guidelines define densitometric osteoporosis based on BMD of the lumbar spine, femoral neck, or total hip without including the radius since it has not been shown that cortical involvement of the radius discriminates well against the risk of fracture in patients with CKD [44]. On the other hand, BMD measurements in the middle third of the radius or in the ultradistal radius can be used to diagnose osteoporosis when the hip and lumbar spine are not assessable [44]. The high interoperative variability in assessing forearm BMD should always be considered. Assessment of the radius should be performed on the arm contralateral to the arteriovenous fistula (AVF), as there may be a local decrease in BMD due to relative immobilization of the AVF arm and/or increased sympathetic tone [45]. Experimental studies also showed growth in bone, which leads to changes in bone structure due to an increase in blood flow.

### 2.3. Limitations of DXA

**Figure 2:**
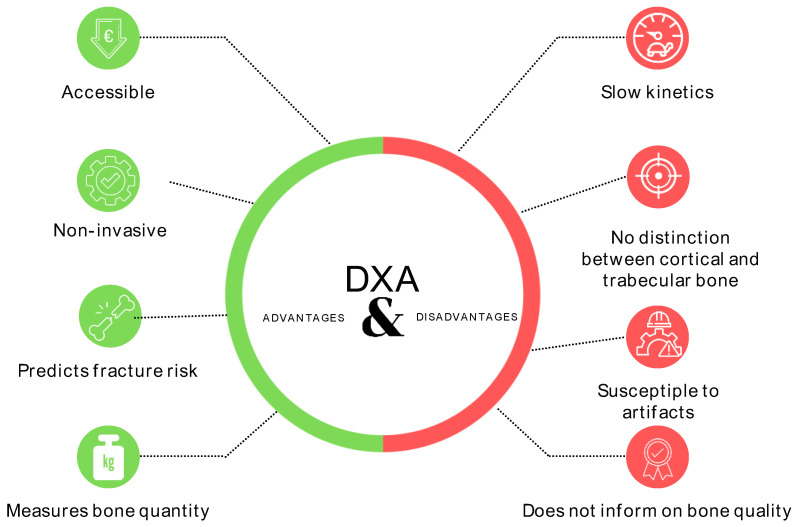
Advantages and disadvantages of DXA [61]. DXA: dual-energy X-ray absorptiometry.

Beyond its inability to differentiate between cortical and trabecular bone, DXA has several limitations (Figure 2). Observational studies demonstrate that most fractures occur in the DXA range of osteopenia and even normal BMD [46,47,48]. In a study of 616 postmenopausal women, only 27% of fractures occurred in women with densitometric osteoporosis (17% had normal BMD, and 57% had osteopenia) [46]. These studies demonstrate that bone fragility depends not only on the amount of bone but also on structural or material properties that are not captured by densitometry, such as trabecular microarchitecture, elasticity, and the quality of the collagen matrix. Patients with secondary osteoporosis or osteoporosis related to metabolic diseases [type 2 diabetes mellitus (DM), Human Immunodeficiency Virus (HIV), hematological diseases, treatment with glucocorticoids, or CKD] in which bone quality is frequently affected, pose a challenge for fracture risk stratification, as assessment based on bone densitometry alone is likely to underestimate fracture risk.

It has been postulated that the presence of aortic calcifications may result in the overestimation of BMD in the lumbar area [49], though direct comparisons of anterior–posterior and lateral lumbar spine BMD indicate that the effect may be minor [50]. Cardiovascular (CV) calcification is a very prevalent condition in all stages of CKD, where, under uremic conditions, an active transformation of vascular smooth muscle cells into osteoblast-like cells occurs [51,52]. CKD patients suffer from an imbalance between inhibitory factors (e.g., pyrophosphates, fetuin-A, osteoprotegerin, Matrix-Gla protein) and promoters [e.g., Ca, P, bone morphogenic protein-2 (BMP-2), BMP-4, RANK-L] of vascular calcification (VC). These factors are related not only to the CV process but also to bone loss, reinforcing the concept that bones and vessels have common metabolic pathways. Bone loss has been associated with the progression of aortic calcifications in the general population [53]; this phenomenon is called the “calcification paradox” [52] and reinforces the idea that bone is an endocrine organ at the heart of CKD-MBD [54]. Some studies have shown an increased incidence of VC in the presence of low bone turnover [55], and an association between the presence of vertebral fractures and VC in HD patients was also observed [56]. Lateral abdominal radiography (thoracolumbar) is a simple and economical method that allows (i) screening for a vertebral fracture that is often asymptomatic, which signals a high risk of new fractures, and (ii) detection and quantification of the presence of VC (Kauppila index) [57,58]. In this context, it may be noted that, parallel to BMD assessment by DXA, the acquisition of a lateral view of the thoracic and lumbar spine (vertebral fracture assessment) should be performed for the diagnosis of vertebral fractures.

Osteoporosis and arthrodegenerative processes frequently coexist in the frail population. Lumbar osteoarthritis or the presence of hip prostheses can give rise to artifacts in the DXA image and represent an additional obstacle when assessing BMD [59,60].

DXA is today’s established standard for assessing BMD according to the current definition criteria for osteoporosis. DXA continues to be the cornerstone for osteoporosis diagnosis, despite its limitations and the emergence of other techniques.

### 2.4. Indications for DXA

European guidance for the diagnosis and management of osteoporosis in postmenopausal women recommends that (where resources for BMD testing are adequate) BMD tests can be undertaken in women with any clinical risk factors [62]. Some national rheumatology guidelines especially recommend densitometry in cases of fragility fractures or the presence of two or more clinical risk factors (Table 1), treatment with aromatase inhibitors, anti-androgenic drugs, or glucocorticoids, and comorbidities associated with secondary osteoporosis [44]. In patients with mild CKD (up to G3a), the management of osteoporosis should follow the recommendations for the general population. For the management of osteoporosis in more advanced stages (G4-5/5D), the European Renal Osteodystrophy Group (EUROD) and the International Osteoporosis Foundation (IOF) published a consensus document recommending that densitometry should be considered in postmenopausal women and patients > 50 years old [63]. Because access to DXA may be unequal, depending on local/regional resources, some authors suggest first calculating fracture risk scores such as FRAX^®^ (Fracture Risk Assessment Tool) score (https://frax.shef.ac.uk/FRAX/tool.aspx?country=9, accessed on 30 December 2023) to decide which patients to proceed with DXA. Patients classified as being at intermediate (FRAX^®^ ≥ 5% for major osteoporotic fracture) or high risk (FRAX^®^ > 3% for hip fracture or ≥7.5% with BMD or ≥10% without BMD for major osteoporotic fracture) would be candidates for further risk reassessment by means of BMD measurement [64]. These cut-off values are not homogeneous across countries, and different recommendations apply. Some national guidelines even advise the initiation of anti-osteoporotic treatment without densitometric evaluation: (i) when FRAX^®^ is ≥3% for hip fracture or ≥10–20% for major osteoporotic fracture; (ii) in patients who have experienced a previous fragility fracture; and (iii) in postmenopausal women and men aged > 50 years who are on long-term corticosteroid treatment or at high doses [62]. However, BMD is not only useful for osteoporosis assessment but also to decide the most appropriate anti-osteoporotic treatment (i.e., BMD <−3 SD or <−3.5 SD indicates a high risk of fracture), to assess the treatment response, and to check adherence (i.e., with denosumab). Of note, another risk calculator, FRAX^®^ plus (https://fraxplus.org/, accessed on 30 December 2023), has recently been released, taking into account additional factors such as the number of falls and recency of an osteoporotic fracture, among others. However, it does not include renal function, CKD stage, or dialysis condition.

Vertebral fracture assessment and/or lateral spine imaging is recommended if there is a history of ≥4 cm height loss, kyphosis, recent or ongoing long-term oral glucocorticoid therapy (equivalent to ≥5 mg prednisone or equivalent per day for ≥3 months), or a T-score ≤ −2.5 SD [62,63].

**Table 1:** Fracture risk factors [64].

FRACTURE RISK FACTORS	
Major (RR > 2)	Minor
BMD T-score ≤ −2.5 SD Age ≥ 65 years Women Previous fragility fracture (spine, hip, wrist) BMI ≤ 20 kg/m^2^ First-degree relative with hip fracture Glucocorticoids (≥5 mg/day of prednisone or equivalent for ≥3 months) ≥2 Falls in the past year	Hyperparathyroidism Eating disorders Chronic malnutrition or malabsorption Hypogonadism or early menopause (40–45 years) Treatment with aromatase inhibitors, gonadotropin-releasing hormone agonists Active smoking Alcohol (>3 U/day) Diabetes mellitus type 1 Rheumatoid arthritis Hyperthyroidism Immobilization

BMD: bone mineral density; BMI: body mass index.

## 3. Bone Quality

### 3.1. Impact of CKD on Bone Quality

**Figure 3:**
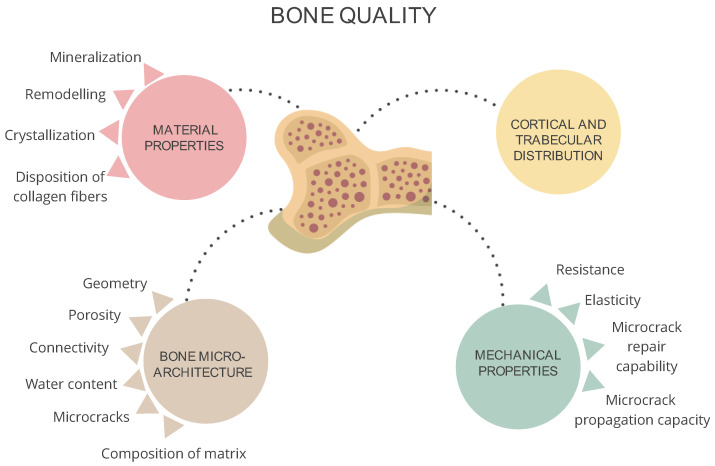
Determinants of bone quality. Adapted from Malluche H. et al., evaluating bone quality in patients with chronic kidney disease [16].

Loss of bone quantity increases the susceptibility to fracture, but this loss of bone mass alone is not sufficient to explain the high incidence of fractures in patients with CKD, suggesting that bone quality also plays an important role in this setting. Bone quality is determined by the material, structural, and mechanical properties of bone, as well as by its capacity to generate and repair bone microdamage (Figure 3).

#### 3.1.1. Uremic Toxins

The bone matrix presents a structure where type I collagen fibers connect with each other to join matrix proteins and crystals that can be mineralized (physiological crosslinks). In uremic conditions, the orientation of the collagen fibers changes, resulting in the establishment of pathological junctions (pathological crosslinks) that lead to the union of immature mineralized crystals [65]. In patients with high bone turnover due to secondary hyperparathyroidism, a lower rate of mineralization of the bone matrix has been described, as well as a lower number of crosslinks of mature collagen fibers [15,66]. These changes in bone properties decrease bone elasticity, which is essential in resisting a fracture after an impact [15]. Elasticity is key to preventing long bone fractures, including femur fractures, which usually occur when force is applied vertically to the bone cortex. Experimental in vivo studies in nephrectomized rats have shown an inverse correlation between creatinine clearance and bone elasticity, measured by dynamic mechanical analysis [67].

It has also been demonstrated that, compared to healthy women, women on dialysis show cortical bone involvement with decreased cortical thickness and increased cortical porosity [68]. Trabecular microarchitecture impairment, with less trabecular bone and greater separation between trabeculae, has also been demonstrated in both men and women with CKD compared with the healthy population [69].

#### 3.1.2. Bone Turnover

The ability to repair the microcracks that occur spontaneously in the bone will also determine its mechanical integrity. Disorders in bone turnover affect bone quality, but via different mechanisms [66]. In patients with low bone turnover, the repair of microcracks may be impaired so that damage accumulates (as during the aging process), resulting in decreased bone strength over time [70]. Compared with patients who have high or normal bone turnover, those with low bone turnover present microstructural alterations such as lower trabecular volume and decreased trabecular thickness. In contrast, patients with high bone turnover present an increase in porosity and thinning of cortices, as well as a decrease in the mineralization ratio of the bone matrix due to the shorter time between remodeling cycles, which prevents complete mineralization. These differences could explain why extra-axial (hip) fractures are more frequent in patients with hyperparathyroidism, while axial (vertebral) fractures may be more frequent in patients with low bone turnover [25]. The relationship between PTH (though not directly a reflection of bone turnover) and the risk of fracture is linear in the early stages of CKD, but at more advanced stages, it becomes an inverted J curve, suggesting that the conditions associated with decreased PTH (malnutrition, inflammation, elderly patients, DM, etc.) could *per se* be the cause of the increased risk of fracture [71]. At the clinical level, however, the relationship between histomorphometric presentation and fracture risk is not completely established. Araújo et al. published a study involving more than 2000 bone biopsies in which no differences in fracture frequency were observed between high and low turnover states; however, mineralization defects were associated with a higher fracture rate [72].

#### 3.1.3. Phosphate Balance

Hyperphosphatemia has been considered a risk factor for osteoporosis, primarily due to the associated increase in PTH [73]. Hyperphosphatemia and high phosphate intake stimulate sclerostin (a potent inhibitor of the Wnt/B-catenin pathway), which inhibits bone formation and mineralization [74]. However, it has also been shown that phosphate is essential for bone mineralization [75]. Kidney transplant recipients who experience transient hypophosphatemia after transplantation have delayed bone mineralization [76]. Intensive dialysis regimens, such as nocturnal hemodialysis, have also been associated with a decrease in phosphate levels that requires discontinuation of phosphate binders and sometimes supplementation with external phosphate in the dialysis fluid [77]. There are even some published cases of severe osteomalacia secondary to chronic hypophosphatemia [78].

#### 3.1.4. Vitamin D Deficiency

Vitamin D deficiency can also cause mineralization deficits [79]. Levels of 25 (OH) vitamin D < 10 ng/mL have been associated with an increased prevalence and incidence of fractures in CKD [80]. The recent update of the Spanish guidelines on the approach to mineral metabolism suggests maintaining calcidiol levels at >20–30 ng/mL, although optimal levels, especially in the case of osteopenia and osteoporosis, would be >30 ng/mL [81], including an appropriate calcium intake [82].

#### 3.1.5. Vitamin K

Vitamin K plays a key role in the carboxylation of various vitamin K-dependent proteins involved not only in blood coagulation but also in bone health, being essential for bone quality. Studies have linked an elevated risk of bone fractures to factors such as insufficient vitamin K intake [83] or low circulating levels of vitamin K [84]. At present, there are no guidelines or recommendations advising on the monitoring or supplementation of vitamin K in patients with CKD.

#### 3.1.6. Systemic Diseases, Comorbidities, and Bone Quality

Some etiologies of CKD *per se* affect bone quality. DM is the most common cause of CKD [85]. Elevated sclerostin levels, accumulation of advanced glycation end-products (AGEs), inflammation, and oxidative stress are possible causes of bone quality impairment in these patients [86]. Some authors suggest that the most important determinant of fracture risk in DM may be the disease itself causing the fracture rather than bone abnormalities, which, if true, would have potential implications for anti-fracture treatment [71].

Autosomal dominant polycystic kidney disease appears to have a bone-specific phenotype characterized by low bone turnover, a better-preserved bone cortex, and elevated sclerostin levels [87]. Polycystins are expressed in multiple tissues and cell types, including osteoblasts and osteocytes, and are involved as mechanosensors in bone [88].

Other inflammatory diseases, such as systemic lupus erythematosus (SLE), show a high rate of vertebral fractures with normal BMD, suggesting an impairment of bone quality [89]. Bone involvement in SLE is multifactorial, with glucocorticoid treatment, inflammatory activity, hormonal disorders, and vitamin D deficiency considered the principal determinant factors [90].

### 3.2. Techniques for Assessing Bone Quality

The limitations of DXA (Figure 2) and recognition of the importance of considering bone quality in the overall assessment of fracture risk, especially in CKD, have prompted the consideration of techniques that can complement DXA. Some are invasive, such as the classic bone biopsy, while others are minimally invasive, e.g., impact microindentation, or non-invasive, e.g., serum bone turnover markers, high-resolution peripheral quantitative computed tomography, TBS, micro magnetic resonance imaging, finite element analysis, Fourier transform infrared spectroscopy, and 3D-DXA [61,91]. Limited attention has been devoted to the assessment of bone quality in patients with CKD, but the scientific evidence has been increasing in recent years [16].

#### 3.2.1. Bone Biopsy

The bone histomorphometric study remains the “gold standard” for the diagnosis and classification of ROD. Classification based on bone turnover, mineralization, and volume (TMV) was described in 2006 with the aim of aiding the interpretation of bone biopsy results with respect to both bone quantity (volume) and bone quality (turnover and mineralization) in patients with ROD [92]. Bone biopsy is the only method available to assess bone mineralization, and this is decisive for the diagnosis of pathologies such as osteomalacia, where DXA will show a low BMD but is otherwise not different from that of osteoporosis with no alteration in mineralization. A bone biopsy will also provide information about the characteristics of the cortical bone (porosity and thickness) and trabecular architecture [93].

Several limitations of the bone biopsy prevent its implementation in routine clinical practice: (i) it is an invasive procedure; (ii) it provides information at a single time point and at a unique site (anterior iliac crest); (iii) pain is a complication and important limiting factor, especially if the technique needs to be performed repeatedly; (iv) the analysis is complex and time-consuming; (v) trained expert personnel, especially for the analysis of the sample. Because of these obstacles, the updated KDIGO guidelines from 2017 accepted that the inability to perform a bone biopsy may not justify withholding antiresorptive therapy from patients at high risk of fracture [13], whereas the same guidelines from 2009 supported bone biopsy in at least five situations: unexplained fractures, persistent bone pain, hypercalcemia or unexplained hypophosphatemia, aluminum toxicity, and prior to initiation of bisphosphonate therapy in patients with chronic kidney disease–mineral bone disorder (CKD-MBD) [10].

Various solutions have been suggested to overcome the above-mentioned limitations, including performing the puncture with a needle of smaller internal diameter (<5 mm rather than 8 mm) that is sufficient for sample extraction while reducing the pain associated with the biopsy [94]. Some authors even propose purely qualitative (rather than semiquantitative) histomorphometric analysis in order to answer three simple questions [93]:

1. What type of turnover is present: low, normal, or high? The answer to this question permits a decision on whether the treatment of osteoporosis should be antiresorptive or anabolic therapy.
2. Is there a mineralization defect? A negative result excludes osteomalacia in a patient with severe bone pain and fractures.
3. Is bone volume low, normal, or high—and is there specific damage to trabecular or cortical microarchitecture? Cortical deficits may support a diagnosis of hyperparathyroidism as the underlying cause of bone fragility.

A recent study based on the Brazilian registry of bone biopsies (REBRABO) showed no association between the type of ROD and hard outcomes such as hospitalization, fractures, or death [95]. Treatment of CKD-MBD is classically considered the first therapeutic step in the treatment of osteoporosis in a patient with CKD, but there is still insufficient evidence to prove that histomorphometric data and ROD type are the main determinants of fracture risk in CKD [96].

Despite their limitations, bone biopsies are re-emerging as a crucial tool in nephrology [97], allowing for a deeper understanding of the effects of the current and more proactive use of anti-fracture treatments in patients with CKD. In this context, although the 2017 KDIGO guidelines do not consider a biopsy mandatory before initiating treatment with an antiresorptive agent [13,81], it is regarded as reasonable to perform one if knowledge of the type of ROD will impact treatment decisions in patients with CKD G3a-5D (not graded) [13,81].

#### 3.2.2. Trabecular Bone Score

Trabecular bone score (TBS) is a textural index determined by analysis of the lumbar DXA image that correlates with the trabecular microarchitecture of the bone. A TBS >1350 indicates that the trabecular microarchitecture is dense and the trabecular structure is well connected (TBS iNsight^TM^ version 2). In the general population, this is a predictor of fracture independent of BMD [98], and it has been incorporated into risk prediction scales such as FRAX^®^. However, TBS is currently used more as an adjuvant to BMD since most clinical practice guidelines do not yet recommend its routine use. Naylor et al. conducted a retrospective study in a cohort of 679,114 adult patients 40 years over, stratified by different estimated GFR, in which they demonstrated that low TBS scores independently associated with a 60% higher risk of suffering a major osteoporotic fracture [99]. However, another recent study with a higher representation of CKD (1624 patients with an estimated GFR between 30–60 mL/min/1.73 m^2^ and 441 with an estimated GFR < 30 mL/min/1.73 m^2^) found that while lower TBS scores were associated with worse kidney function, the addition of TBS to the FRAX^®^ score with BMD did not significantly improve fracture risk prediction [100].

Other studies have found TBS to be useful in predicting fracture risk in renal transplant and HD patients, although data are limited [101,102]. Lower TBS has also been associated with a higher number of cardiovascular events and higher mortality in HD patients, suggesting that it could be an indicator of frailty in this population [102].

#### 3.2.3. High-Resolution Peripheral Quantitative Computed Tomography

High-resolution peripheral quantitative computed tomography (HR-pQCT) is a technique that analyzes bone microstructure in a non-invasive manner. It is important to note that HR-pQCT captures images of the distal radius or distal tibia, whereas a bone biopsy obtains material from the iliac crest. These bones have different mechanical and metabolic characteristics, and as a result, comparisons of the techniques can yield only modest correlations [103]. HR-pQCT has a resolution of approximately 80 µm and allows 3D imaging and detailed examination of the trabecular compartment and cortical porosity, as well as the volumetric parameters of bone mass. HR-pQCT and magnetic resonance imaging (MRI) have been postulated to be superior to DXA in discriminating fractures in patients with CKD, but the evidence is still contradictory [104,105]. Importantly, the high cost of these imaging techniques has prevented extensive use in clinics, limiting their use to research purposes.

#### 3.2.4. Impact Microindentation

Impact microindentation (IMI) is a novel technique designed to assess bone strength from a global perspective and in a minimally invasive way, providing information on both bone quality and bone quantity. It is based on the principle that the depth of penetration of a micron-sized probe into the bone surface, with local reproduction of microcracks by separation of microfibers of mineralized collagen, reflects the resistance of the bone to fracture after mechanical impact. The technique represents the possibility of directly assessing the mechanical characteristics of cortical bone in vivo [106,107].

**Figure 4:**
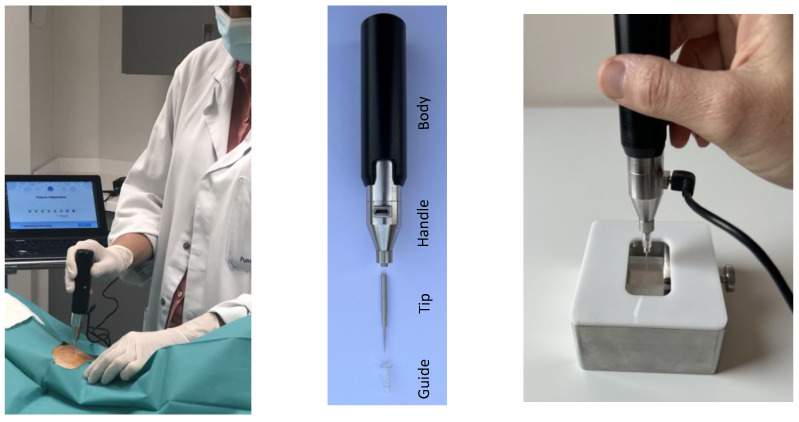
Hand microindenter (Osteoprobe^®^).

Osteoprobe^®^ [108] is a portable, hand-held device for performing IMI (Figure 4). The device expresses the bone strength result as BMSi (Bone Material Strength index), which represents the ratio between the distance the needle probe penetrates the bone (anterior tibial face) and the distance it penetrates a reference standard (a methyl methacrylate phantom). Tolerance and acceptance by patients are excellent, and the complication rate is minimal, allowing iterative exploration [109,110,111].

Recent studies indicate that IMI assesses the properties of subperiosteal bone material, but it is still unknown exactly what properties relate to the bone quality it is measuring. It has been found that local mineral content, nanoporosity, and pyridinoline content at the subperiosteal level in the transiliac bone biopsy are related to BMSi values measured in the tibia [112]. It has also been observed that parameters related to the quality of the organic matrix content (mineral-to-matrix ratio) are strongly associated with BMSi (*r* = 0.735; *p* = 0.0063) [113].

In a study comparing the behavior of BMSi in patients with and without fractures (normal kidney function), BMSi values were lower in patients compared to those without fragility fractures, despite similar BMD. IMI could be especially useful in patients with secondary osteoporosis and metabolic bone disorders (e.g., those with CKD), in which BMD is not the sole determinant of bone strength. Thus, previous studies have evaluated IMI in patients with DM [114], hyper- and hypothyroidism [115,116], acromegaly [117], HIV [118], monoclonal gammopathy of undetermined significance (MGUS) [119], and CKD. Pérez-Sáez et al. previously published a cross-sectional study to characterize bone health by means of BMD, TBS, and IMI in patients with kidney failure at the time of kidney transplantation, compared to a healthy population. Patients with kidney failure had lower bone mass, a worse bone trabecular index, and a lower resistance index measured by IMI [120]. In addition, the impact of corticosteroids on bone resistance, measured by IMI, has been evaluated in kidney transplant recipients [121].

## 4. Future Directions

CKD is more prevalent in older individuals, women, and patients with diabetes mellitus (a major risk factor for fracture), and the use of glucocorticoids is not infrequent in some kidney diseases [4]. Therefore, nephrology societies are becoming increasingly aware of the issue of bone fragility in our patients, and several initiatives emphasize this serious problem. The scenario in the approach to osteoporosis in patients with CKD has undergone significant changes in recent years:

1. Several studies have shown that DXA can predict fracture risk in patients with CKD [11,12].
2. KDIGO guidelines no longer consider it mandatory to perform a bone biopsy before initiating anti-fracture therapy [13].
3. There is growing evidence with regard to the safety and efficacy of bone-targeting drugs in the setting of (advanced) CKD, thereby reducing concerns about the safety of these therapies.

These developments should encourage nephrologists to be more proactive in the assessment of fracture risk. Such assessment should be systematically included in routine clinical practice, with adaptation according to the resources of the local setting. The development of new techniques to assess bone quality may increase our understanding of bone behavior in patients with CKD and improve the overall detection of imminent risk of fracture (within 1–2 years) to offer patients the best and earliest possible treatment. In the future, new bone biomarkers (including omics) [122] may, in a more affordable way, not only offer guidance in the choice of the best treatment for osteoporosis but also allow the monitoring of treatment efficacy. Nevertheless, all of these techniques are not universally accessible, and, in certain instances, their high costs may limit their implementation in clinical practice. Finally, in most patients with CKD, hip fractures occur because of a fall, and falls are especially frequent in patients receiving dialysis [123]. It is then important to consider and address not only bone strength (quality and quantity) but also all the phenomena associated with a risk of falling (e.g., sarcopenia, atrophy and muscle strength, loss of vision and equilibrium, hypotension, use of neurotropic or sedating medications) in order to prevent fractures.

## 5. Conclusions

Osteoporosis is a state of bone fragility that implies a reduction in resistance to bone fracture after trauma. Resistance, especially in CKD, is determined not only by bone quantity but also by bone quality, which is adversely affected by uremia, ROD, and metabolic acidosis, among other factors. The high incidence of fractures and their impact on morbidity and mortality must be an impetus to improve the assessment of fracture risk in CKD. Techniques for the evaluation of bone quality, such as TBS, HR-pQCT (trabecular microarchitecture, cortical porosity, and volumetric parameters of bone mass), bone biopsy (bone volume, mineralization, and turnover), and IMI (bone strength), may prove complementary to the assessment of BMD by DXA for the assessment of fracture risk in patients with CKD at the individual level.
